# Premedication of Pediatric Cardiac Population with Midazolam: Comparison of Oral and Sublingual Administration Regarding Plasma Midazolam Concentration, Clinical Effectiveness, Hemodynamic and Behavioral Outcomes

**DOI:** 10.3390/children13081067

**Published:** 2026-08-11

**Authors:** Theofili Kousi, Afroditi Karafotia, Vlasios Karageorgos, Georgios Gkantinas, Ioanna Sofianidou, Meletios Kanakis, Alexandra Smina, Ioanna Zergioti, Constantin Tamvakopoulos, Theofani Antoniou

**Affiliations:** 1 Department of Anesthesiology, Onassis Hospital Athens, 17674 Athens, Greece; a.karafotia@onasseio.gr (A.K.); v.karageorgos@onasseio.gr (V.K.); gadinasgio@gmail.com (G.G.); i.sofianidou@onasseio.gr (I.S.); t.antoniou@onasseio.gr (T.A.); 2 Department of Congenital Cardiac Surgery, Onassis Hospital Athens, 17674 Athens, Greece; m.kanakis@onasseio.gr; 3 Center for Clinical, Experimental Surgery and Translational Research, Pharmacology-Pharmacotechnology, Biomedical Research Foundation, Academy of Athens, 11527 Athens, Greece; smina.alexandra@gmail.com (A.S.);; 4 School of Applied Mathematical and Physical Sciences, National Technical University of Athens, Heroon Polytehneiou 9, 15780 Athens, Greece; zergioti@central.ntua.gr

**Keywords:** midazolam, sublingual, oral, pediatric anesthesia, congenital heart disease, cardiac surgery, premedication, pharmacokinetics, hemodynamic stability

## Abstract

**Highlights:**

**What are the main findings?**

- Lower-dose sublingual midazolam (0.3 mg/kg) produced no statistically significant differences in adjusted plasma midazolam or 1-OH-midazolam concentrations, behavioral outcomes, HR, or SpO_2_ compared with oral midazolam (0.5 mg/kg).

**What are the implications of the main findings?**

- Sublingual midazolam may represent a feasible lower-dose alternative for premedication in children with congenital heart disease.
- The results support further development of acceptable, child-friendly sublingual formulations, although equivalence cannot be concluded from this study.

**Abstract:**

**Background:** Midazolam is widely used as a pediatric premedication, but evidence from direct comparisons of oral and sublingual administration in children with congenital heart disease remains limited, particularly that from pharmacokinetic and physiologic data analyzed together. **Methods:** We conducted a single-center prospective randomized study comparing oral midazolam 0.5 mg/kg with sublingual midazolam 0.3 mg/kg in children undergoing cardiac surgery or catheterization procedures under general anesthesia. Plasma midazolam and 1-hydroxymidazolam concentrations were measured approximately 30 min after administration. Log-transformed concentrations were compared using regression/ANCOVA models adjusted for dose and age. Changes in mean arterial pressure (MAP), heart rate (HR), and oxygen saturation (SpO_2_) were analyzed from baseline to 15 and 30 min. Behavioral outcomes included the sedation score, separation from parents, and mask acceptance. **Results:** Sixty-eight children were randomized; 65 had evaluable pharmacokinetic samples and formed the complete-case pharmacokinetic cohort. Adjusted plasma midazolam concentrations did not differ significantly between the groups, with an adjusted geometric mean ratio for sublingual versus oral administration of 0.98 (95% CI 0.53–1.79; unadjusted *p* = 0.940; Holm-adjusted *p* = 1.000). The corresponding ratio for 1-hydroxymidazolam was 1.37 (95% CI 0.55–3.41; unadjusted *p* = 0.494; Holm-adjusted *p* = 1.000). HR and SpO_2_ changes were non-significant between the groups. At 30 min, sublingual administration was associated with a lower adjusted change in MAP compared with oral administration (adjusted difference −12.08 mmHg, 95% CI −19.74 to −4.42; unadjusted *p* = 0.002, Holm-adjusted *p* = 0.012). Behavioral outcomes did not differ significantly between the groups. **Conclusions:** In this prospective randomized pediatric cardiac cohort, oral midazolam 0.5 mg/kg and sublingual midazolam 0.3 mg/kg produced comparable plasma concentrations and similar behavioral outcomes. Sublingual administration was not associated with worse HR or SpO_2_ responses, although an isolated lower MAP change at 30 min warrants confirmation in larger studies. Sublingual midazolam may represent a feasible lower-dose alternative for premedication in this population.

## 1. Introduction

Hospitalization and surgery can be a source of stress and anxiety for pediatric patients [1]. Induction of anesthesia is often highly distressing, and studies have shown that an anxious child during anesthetic induction has a greater possibility of developing adverse clinical outcomes, such as emergence delirium and negative behavioral changes, namely, separation anxiety and aggression [2,3,4].

Children with cardiac disease may experience elevated preoperative anxiety because of previous hospitalizations, operations, or catheterization laboratory interventions. Parental stress related to the severity of the child’s condition and the planned intervention may further amplify the child’s anxiety [5,6].

Non-pharmacological strategies for reducing preoperative anxiety and improving anesthesia compliance in children have evolved [7]. Virtual reality acts through cognitive distraction and immersion and has shown promising results regarding better preoperative anxiety scores and improved cooperation during anesthesia induction as children become more familiarized with the whole process. However, these approaches can be costly, may be less suitable for children younger than 5 years, and appear more likely to reduce than fully replace sedative requirements [8,9].

Among the pharmacological options for reducing anxiety prior to surgery in pediatric populations, midazolam remains one of the most widely used premedication agents because of its anxiolytic, sedative, and amnestic properties [10]. Its cardiovascular stability makes it attractive for the pediatric cardiac population, which is more vulnerable to changes in myocardial contractility, vascular tone, heart rate, and oxygenation, particularly when intracardiac or extracardiac shunts are present [11].

The oral route is well established and widely utilized in clinical practice, but sublingual administration may offer practical advantages, including faster mucosal absorption and partial avoidance of first-pass metabolism [12]. Prior pediatric studies have suggested that sublingual midazolam is at least comparable with oral midazolam, and there are some reports that it may produce earlier or deeper sedation. Kattoh et al. reported similar efficacy of sublingual and oral midazolam in pediatric anesthesia, whereas Gupta et al. found higher sedation scores with the sublingual route [13,14]. In parallel, the earlier cardiac surgery literature supports oral midazolam as a safe premedication option in children with congenital heart disease.

Despite these data, studies in the literature directly combining route comparisons with measured plasma concentrations, metabolite analysis, and serial physiologic observations in a pediatric cardiac cohort are sparse. The present study was designed to compare oral midazolam 0.5 mg/kg with sublingual midazolam 0.3 mg/kg across three complementary domains: plasma pharmacokinetic measurements, hemodynamic and oxygenation stability, and peri-induction behavioral performance.

## 2. Methods

### 2.1. Study Design and Approval

This was a single-center prospective randomized study. The prespecified primary outcome was a between-route comparison of plasma midazolam concentrations, adjusted for the administered dose and age. Plasma 1-hydroxymidazolam concentrations; changes in mean arterial pressure (MAP), heart rate, and oxygen saturation; and behavioral outcomes (sedation, parental separation, and mask acceptance) were prespecified secondary outcomes and are reported as exploratory.

The study protocol was approved by the Ethics Committee and the Scientific Board of the Onassis Hospital (B.03. Π.Ε.Ε 709 (ΙΩ Γ 010)/25 February 2021); written informed consent was obtained from the parents or legal guardians of all participants. The trial has been registered in the International Standard Randomised Controlled Trial Number (ISRCTN) registry (ISRCTN33764131—https://doi.org/10.1186/ISRCTN33764131) and was conducted between July 2021 and October 2022. It was registered retrospectively in April 2026 due to an administrative oversight following changes to the original study design prior to its conduct. The original study protocol also included an experimental component evaluating oral and sublingual administration of midazolam using 3D-printed, dose-individualized films in mice. However, despite several attempts, technical difficulties meant that it was not feasible to manufacture the 3D-printed films using the liquid midazolam formulation available for this study. Consequently, the experimental component was omitted, and we decided to proceed with only the clinical component of the study, which was completed according to the initial planning. As a result, the trial was registered retrospectively. The outcomes and statistical analyses reported in the manuscript were prespecified before data analysis. The initial study plan included comparing the behavioral responses and plasma drug levels between the groups, with the intention of subsequently performing the same comparisons in experimental animals receiving premedication either orally or via a 3D-printed sublingual midazolam film.

### 2.2. Patient Recruitment

Children scheduled for cardiac surgery or catheterization laboratory interventions under general anesthesia were screened after standard preoperative anesthetic evaluation. Their parents or legal guardians were informed about the purpose and procedures of the study.

Inclusion criteria:

1. Age 6 months to 8 years;
2. Diagnosed heart disease;
3. Undergoing general anesthesia for pediatric cardiac surgery;
4. Undergoing general anesthesia for interventions in the catheterization laboratory.

Exclusion criteria:

1. Body weight less than 5 kg or >40 kg;
2. History of adverse or allergic reaction to midazolam;
3. Conditions in which sedative premedication may be contraindicated (anticipated difficult airway, increased risk of aspiration, acute systemic illness, upper respiratory tract infection).

### 2.3. Randomization and Blinding

Patients were randomized to the oral or sublingual midazolam group using a computer-generated allocation sequence. Premedication was administered by a nurse who was not involved in outcome assessment. Because oral and sublingual placements are visibly distinguishable, neither the administering clinician nor the parents could be blinded to the physical route. However, parents were unaware of the administered dose and of the direction of the study hypothesis. The anesthesiologist who performed perioperative evaluations and managed the intraoperative anesthetic was blinded to the dose and route of premedication.

### 2.4. Anesthetic Management

Preoperative fasting was applied as per local protocol.

Thirty minutes before arrival in the operating theater, the children received premedication according to their randomized group allocation: midazolam 0.5 mg/kg orally or midazolam 0.3 mg/kg sublingually. The oral midazolam dose of 0.5 mg/kg was selected because it represents the most widely accepted and extensively studied regimen for pediatric preoperative anxiolysis, providing effective sedation with an acceptable safety profile across a broad age range [15]. The sublingual dose of 0.3 mg/kg was chosen based on published pediatric studies demonstrating its clinical efficacy for premedication [13,16] and the pharmacokinetic rationale that sublingual administration partially bypasses intestinal and hepatic first-pass metabolism, resulting in greater systemic drug exposure and a more rapid onset of action than oral administration [17]. Although no validated oral-to-sublingual dose conversion ratio for midazolam has been established in children, the selection of a lower sublingual dose was intended to account for its higher bioavailability while remaining within the range of doses. This dosing approach has also been used in previous comparative studies [18,19].

Because an injectable midazolam formulation with a bitter taste was used, an equal volume of flavored paracetamol syrup was added for oral administration, and an equal volume of 10% dextrose in water was added for sublingual administration. The administration techniques intentionally differed between the groups. In the oral group, the study mixture was administered using a small cup or, in younger children, via syringe onto the dorsum of the tongue to facilitate immediate swallowing and gastrointestinal absorption. In the sublingual group, the study mixture was administered using a syringe placed beneath the tongue to maximize contact between the drug and the sublingual mucosa and thereby facilitate transmucosal absorption. In older children, specific instructions were given to keep the medication under the tongue and to avoid swallowing for as long as possible. Administration was performed by trained study personnel who visually confirmed sublingual placement. Nevertheless, partial swallowing cannot be excluded.

The children were monitored throughout the period after premedication administration. Blood pressure, heart rate, and oxygen saturation (SpO_2_) were recorded at baseline and at 15 and 30 min after drug administration. Blood pressure values were recorded either as systolic (SAP)/diastolic (DAP)/mean (MAP) arterial pressures or as systolic/diastolic only. When the mean arterial pressure was not explicitly recorded, MAP was calculated as DAP + (SAP − DAP)/3.

Sedation was assessed at 15 and 30 min after drug administration using a five-category sedation score: A = anxious, B = restless, C = calm and awake, D = sedated but easily woken, and E = deeply sedated. A positive sedation outcome was predefined as C or D at each time point.

Separation from parents was assessed at approximately 30 min after drug administration using a three-category score: A = easy, B = mild resistance, and C = strong resistance. A positive separation outcome was predefined as A.

Mask acceptance was assessed at approximately 30 min after drug administration using the same three-category score: A = easy, B = mild resistance, and C = strong resistance. A positive mask acceptance outcome was predefined as A.

On arrival in the operating theater, standard monitoring was established, including electrocardiography, pulse oximetry, and noninvasive blood pressure monitoring until placement of an arterial catheter, when applicable. Venous access was secured, and blood samples for pharmacokinetic assessment of plasma midazolam and 1-hydroxymidazolam concentrations were collected approximately 30 min after drug administration.

The attending anesthesiologist who performed and recorded all measured clinical parameters was blinded to the route of midazolam administration. The 15 min evaluation was performed in the patient room. The child was then transferred under monitoring to the operating theater. On arrival in the pre-anesthesia area, the child was separated from their parents and transferred to the operating room. Once monitoring was connected, hemodynamic and oxygenation parameters were recorded, a mask was applied, and anesthesia induction was initiated. Blood sampling was performed after the child had been put to sleep using inhalational anesthesia. The aforementioned sequence of events coincided with our 30 min measurement timeframe.

### 2.5. Single-Time-Point Plasma Concentration Assessment

The 30 min blood sampling time point for the measurement of plasma midazolam concentrations was selected to coincide with the clinical evaluation, behavioral assessment, and measurement of hemodynamic variables at the time of anesthesia induction, allowing simultaneous pharmacokinetic and clinical assessment. This approach also enabled an evaluation of whether potential differences in plasma midazolam concentrations were associated with corresponding changes in behavioral or physiological parameters.

Midazolam is a controlled substance classified in Table D of the Individual Listing for Greece according to the Code of Laws for Narcotic Drugs (Law 3459/2006). Due to these regulatory restrictions, Dormixal 15 mg/3 mL injectable solution was used for midazolam LC-MS/MS method development in this study. 1-Hydroxymidazolam 5 mg was purchased from Cayman Chemicals (Ann Arbor, MI, USA). The LC-MS-grade solvents ammonium acetate, formic acid (FA), and water (H_2_O) were procured from Fisher Scientific (Loughborough, UK). Acetonitrile (ACN; LC-MS grade) was bought from Carlo Erba (Milan, Italy).

Dormixal 15 mg/3 mL was diluted into acetonitrile–water (ACN:H_2_O) to prepare a 1 mg/mL stock solution. Following this, midazolam standards in the concentration range of 1 to 200 ng/mL were prepared via dilution in ACN:H_2_O. 1-Hydroxymidazolam (item 10,385.5 mg) was dissolved in 1 mL of solvent, and metabolite standard solutions were prepared. All stock and working solutions were stored at 4 °C until the day of sample preparation in human plasma.

*Detection and quantification of midazolam and 1-hydroxymidazolam via LC-MS/MS*

The levels of midazolam and its metabolite 1-hydroxymidazolam in standard and unknown samples were detected and quantified via LC-MS/MS. A Triple Quad 5500+ LC-MS/MS System—QTRAP (AB SCIEX LLC, Redwood City, CA, USA) was used for method development. A dC18 column (Waters, Atlantis, 2.1 × 50 mm, 3 μΜ) was used for chromatographic separation of midazolam and its metabolite at a flow rate of 0.3 mL/min. The mobile phase consisted of solutions A (90% H_2_O, 10% ACN, 0.1% formic acid [FA], and 2 mM ammonium acetate) and Β (10% H_2_O, 90% ACN, 0.1% formic acid [FA], and 2 mM ammonium acetate) and needle wash (ACN:H_2_O 1:1), and the injection volume for each sample was 10 μL. The analytes of interest (*midazolam and 1-hydroxymidazolam*) were eluted separately via the implementation of an 8.5 min gradient system described accordingly: 80% A and 20% Β (0.0–0.5 min), 20% A and −70% Β (2.5–5.00 min), and 80% A and 20% Β (6.0–8.5 min). The transitions 326.1/209.1 for *midazolam*, 342.0/324.0 for the primary metabolite 1-OH midazolam, and 309.0/163.0 for warfarin (IS) were monitored via multiple reaction monitoring at times of 2.3, 2.2, and 3.5 min, respectively. The mass spectrometry (MS) system was operated using positive electrospray ionization mode (ESI). The LC-MS/MS methodology and linearity were set up using human plasma with concentrations between 1 and 200 ng/mL for both analytes.

For standard curve sample preparation, 50 μL of human plasma was spiked with 50 μL of *midazolam* and 50 μL of *1-hydroxymidazolam* standard solution. A volume of 50 μL of warfarin was added for a final concentration of 10 ng/mL in human plasma. Accordingly, 50 μL aliquots of unknown samples (pediatric plasma) were prepared with a 10 ng/mL IS concentration. Double-blank (human plasma in ACN:H_2_O) and blank IS (10 ng/mL) samples were included to determine the noise levels of the assay. The samples were centrifuged at 13.000 rpm for 15 min following protein precipitation with cold ACN (400 μL). The supernatants were transferred into glass tubes, and the samples were evaporated to dryness at 50 °C for approximately 1 h. The samples were reconstituted in 150 μL of 80% A–20% B solution, vortexed, and added to 96-well plates for LC-MS/MS analysis.

### 2.6. Statistics and Sample Size

Continuous variables are presented as means ± SDs and medians with interquartile ranges (IQRs). Because the midazolam and 1-hydroxymidazolam concentrations were right-skewed, pharmacokinetic comparisons were performed using linear regression/ANCOVA on natural log-transformed concentrations, adjusted for the administered dose in mg/kg and age. The results are reported as adjusted geometric mean ratios with 95% confidence intervals (CIs).

Hemodynamic and oxygenation outcomes were analyzed as changes from baseline at 15 and 30 min using linear regression adjusted for age and body weight. Behavioral outcomes were compared using Fisher’s exact test.

To account for multiple hypothesis testing, *p*-values were adjusted using the Holm–Bonferroni step-down procedure, which controls the family-wise error rate. Global correction was applied across the 12 reported between-group outcome comparisons, comprising two plasma concentration outcomes, six physiologic outcomes, and four behavioral outcomes. Unadjusted and multiplicity-adjusted *p*-values are reported. Statistical significance after correction was defined as an adjusted *p*-value of <0.05. Effect estimates and 95% confidence intervals are presented without multiplicity adjustment.

The planned sample size was 60 children, with 30 per group, providing approximately 80% power to detect a standardized between-group difference of about 0.7. The final pharmacokinetic complete-case cohort included 65 children, preserving similar power for moderate-to-large continuous effects.

## 3. Results

### 3.1. Baseline Characteristics

**Figure 1:**
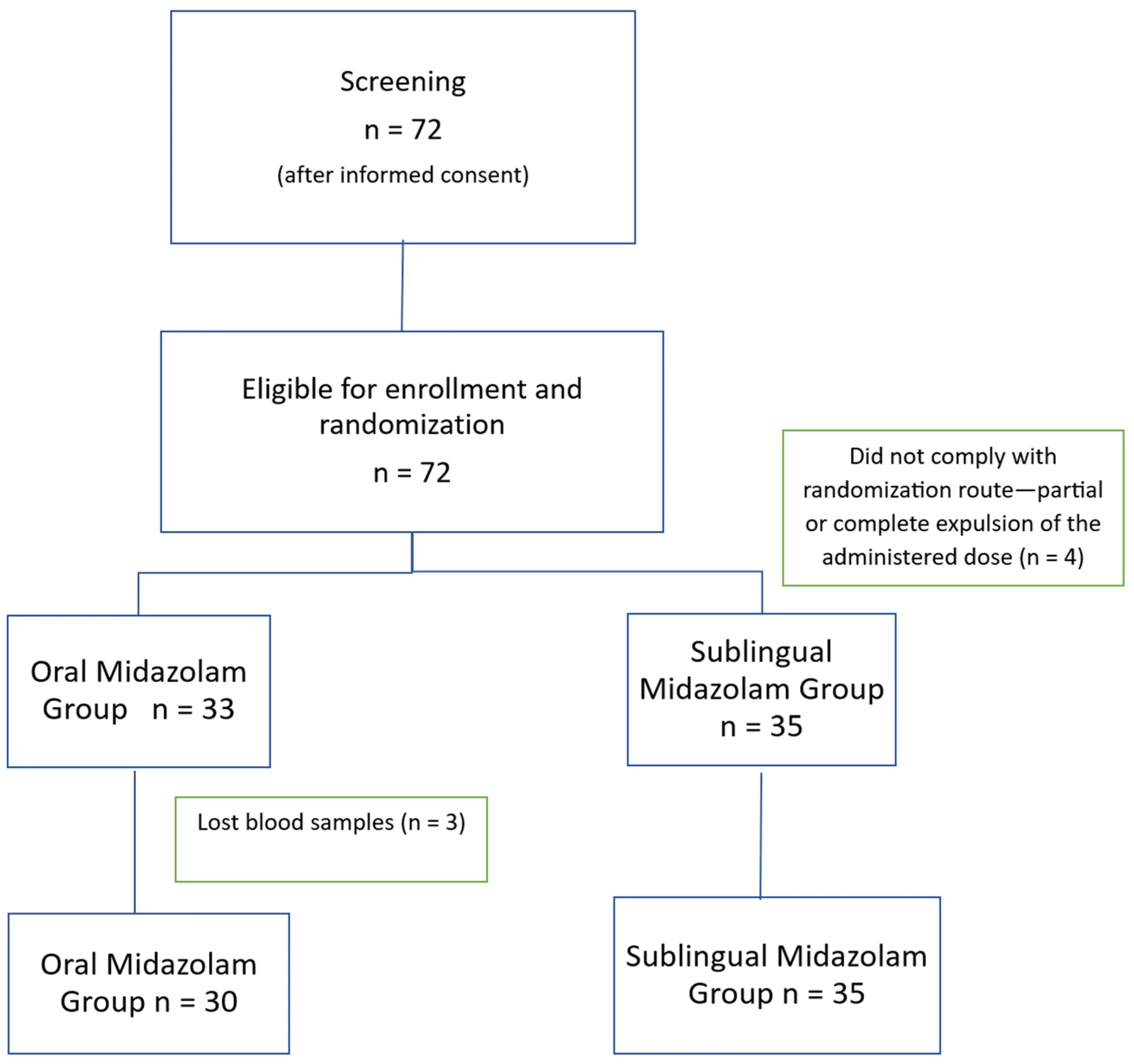
CONSORT flowchart.

Because four children expectorated the medication and did not comply with the administration route, sixty-eight children were included after randomization: 33 for oral midazolam and 35 for sublingual midazolam. Three blood samples from the oral midazolam group were inappropriate for analysis, leaving 65 observations for the complete-case pharmacokinetic analysis (Figure 1). The baseline demographic characteristics and procedure types performed are summarized in Table 1 and Table 2.

### 3.2. Pharmacokinetic Outcomes

**Figure 2:**
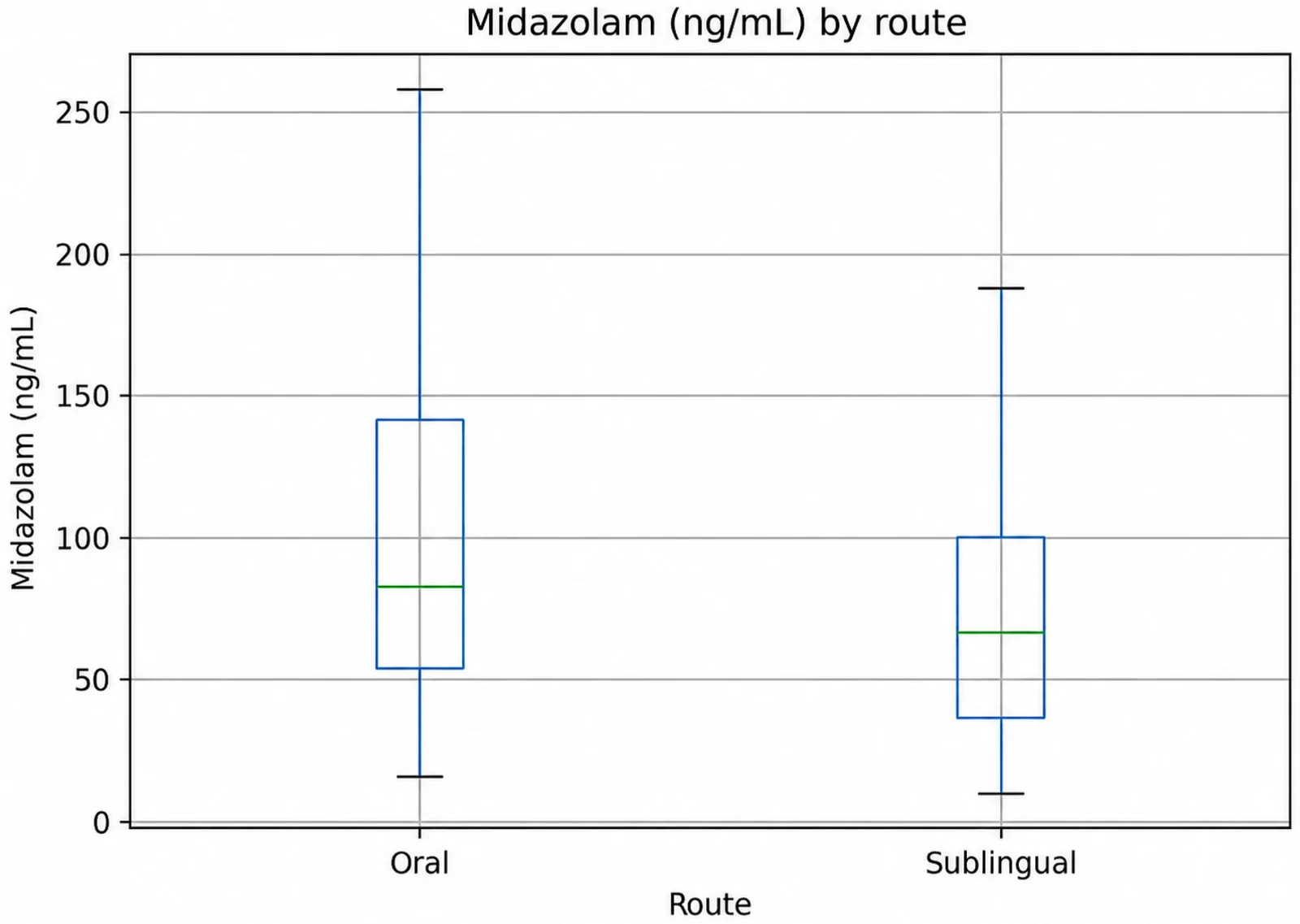
Plasma midazolam concentrations by administration route.

**Figure 3:**
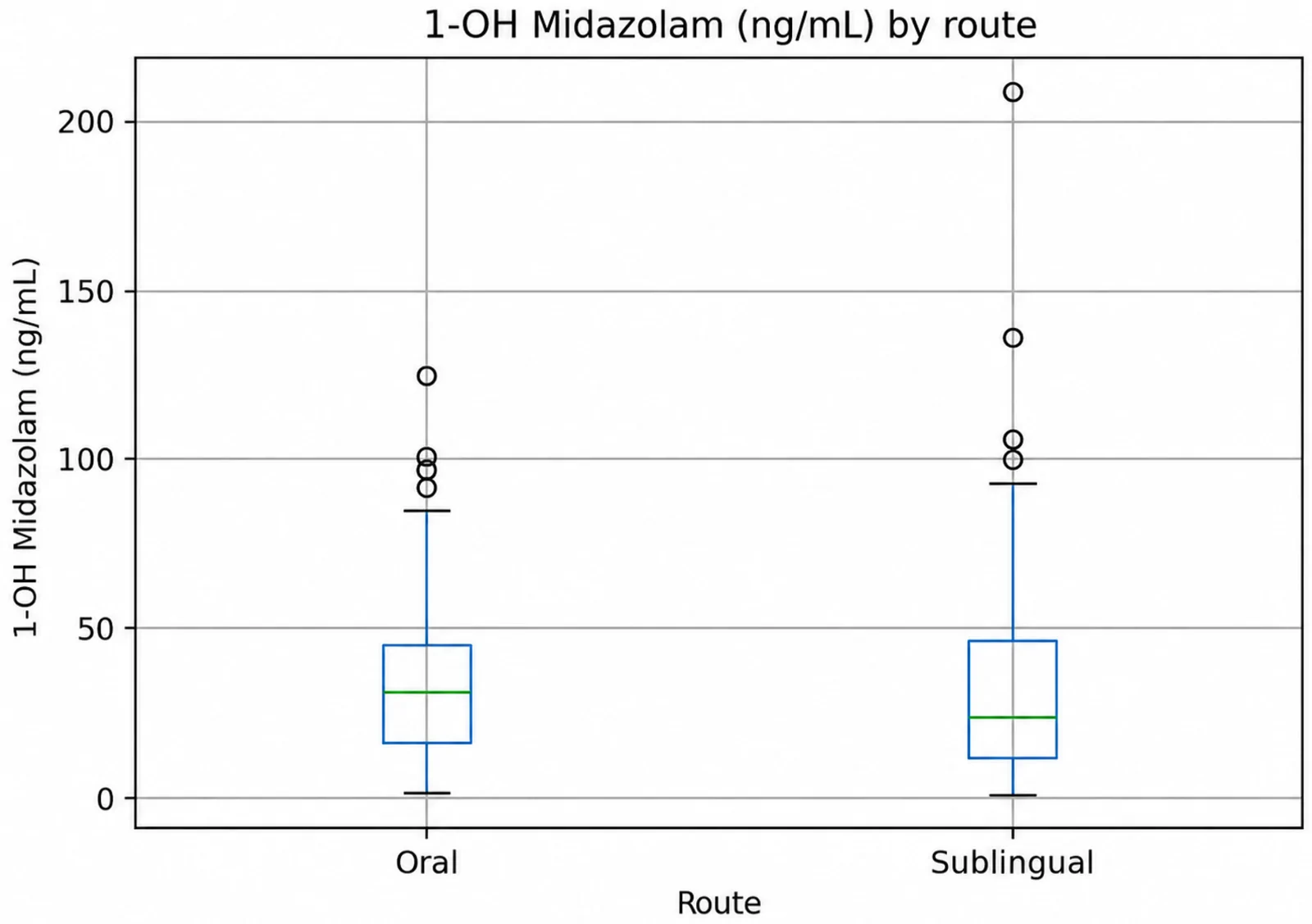
Plasma 1-hydroxymidazolam concentrations by administration route.

Table 3 shows that after adjustments for dose and age, there was no evidence of a route effect for the midazolam concentration (Figure 2) (adjusted geometric mean ratio S/O 0.98, 95% CI 0.53–1.79; unadjusted *p* = 0.940; Holm-adjusted *p* = 1.000) or for the 1-hydroxymidazolam concentration (Figure 3) (ratio 1.37, 95% CI 0.55–3.41; unadjusted *p* = 0.494; Holm-adjusted *p* = 1.000).

### 3.3. Hemodynamic and Oxygenation Outcomes

Route–dose comparisons for physiologic outcomes, summarized in Table 4, were based on changes from baseline (Δ at 15 and 30 min). Absolute values are presented in Table S1 of the Supplementary Materials.

No regimen-associated difference was observed for ΔHR or ΔSpO_2_ at either time point. For MAP, the 15-min adjusted difference did not reach significance (S−O −6.08 mmHg, 95% CI −13.57 to 1.40; *p* = 0.109). At 30 min, however, the sublingual group showed a lower adjusted ΔMAP than the oral group (−12.08 mmHg, 95% CI −19.74 to −4.42; unadjusted *p* = 0.002, Holm-adjusted *p* = 0.012).

### 3.4. Behavioral Outcomes

Behavioral outcomes are summarized in Table 5. At 15 min, a positive behavioral score (calm/awake or easily rousable) was observed in 80.0% of children receiving oral midazolam and 85.7% receiving sublingual midazolam (*p* = 0.742). Positive sedation ratings were similarly frequent in the two groups (86.7% vs. 88.6%, *p* = 1.000). Easy separation from parents occurred in 83.3% of oral cases and 85.7% of sublingual cases (*p* = 1.000). Easy mask acceptance was observed in 43.3% and 54.3% of the children, respectively (*p* = 0.459), showing a non-significant tendency in favor of the sublingual group. Overall, no significant differences in behavioral outcomes (Holm-adjusted *p* = 1.000) were detected between the administration routes, although the available sample size was underpowered to exclude small-to-moderate differences in binary behavioral outcomes.

## 4. Discussion

This prospective randomized analysis integrated plasma concentrations with physiologic and behavioral data to compare oral midazolam 0.5 mg/kg with sublingual midazolam 0.3 mg/kg in children undergoing cardiac surgery or catheterization laboratory procedures. The principal findings were that, first, plasma midazolam and 1-hydroxymidazolam concentrations did not differ significantly between the routes after adjustments for dose and age; second, HR and SpO_2_ changes showed no statistically significant difference between the groups; third, behavioral outcomes also showed no statistically significant difference; and fourth, an isolated signal of lower adjusted ΔMAP at 30 min was observed in the sublingual group.

The comparable plasma midazolam and 1-hydroxymidazolam concentrations suggest that within the regimens used in this cohort, sublingual administration did not produce clearly higher or lower systemic exposure than oral administration once dose differences were taken into account. This is clinically relevant because the oral group generally received higher mg/kg doses than the sublingual group, yet the adjusted models still showed overlapping exposure estimates.

The behavioral results align more closely with a study by Kattoh et al. [13], which found comparable efficacy of oral and sublingual midazolam, than with a study by Gupta et al. [14], which reported better sedation with the sublingual route. The discrepancy in Gupta’s study may reflect differences in dosing strategies, as equivalent doses were administered via both routes despite their distinct bioavailability profiles.

The present dataset did not demonstrate statistically significant differences for either administration regimen regarding calmness at 15 min, overall sedation rating, separation from parents, or mask acceptance. These null behavioral comparisons should be interpreted as an absence of evidence rather than evidence of absence: this study was underpowered to detect small-to-moderate differences in the categorical behavioral outcomes (calmness, sedation rating, parental separation, and mask acceptance), and type II error cannot be excluded.

Achieving comparable behavioral and sedation scores with a lower dose of midazolam administered via the sublingual route may confer additional clinical benefits. Paradoxical agitation with midazolam, including disinhibition, restlessness, hallucinations, and disorientation, is uncommon but distressing. The reported incidence of this reaction is 1–10%, and it is usually observed in younger children and elderly patients. Paradoxical agitation is an idiopathic reaction, and various studies support its reversal with flumazenil. Additional doses of midazolam may further enhance this phenomenon and should be avoided [20]. Even though we have come across such reactions in our clinical practice, none of our studied pediatric patients exhibited them. The lower doses used in the sublingual route might be favorable in this context, as young age and higher dosage are recognized as risk factors [21]; however, our study was not designed or powered to evaluate this outcome.

We did not assess delayed recovery or postoperative delirium, and a considerable proportion of children in this pediatric cardiac cohort remained intubated and required further sedation in the pediatric intensive care unit. Therefore, no conclusion can be drawn regarding whether the lower sublingual dose influenced recovery or delirium. Garcia et al. reported no dose–response relationship between oral midazolam doses and emergence or discharge times in a retrospective pediatric tonsillectomy and adenoidectomy cohort [22]. Earlier prospective day-case studies using oral midazolam 0.5 mg/kg demonstrated delayed early recovery without prolonged discharge times [23,24].

From a safety perspective, the absence of a route-associated HR or SpO_2_ signal is reassuring in this pediatric cardiac cohort. It is consistent with earlier studies in the literature supporting the safety of oral midazolam in children with congenital heart disease undergoing cardiovascular surgery [11].

The statistically significant between-group difference in MAP at 30 min warrants cautious interpretation, as the absolute values remained age-appropriate, no patient developed clinically significant hypotension or required hemodynamic intervention, and the finding was isolated, with no parallel changes in behavioral scores, heart rate, oxygen saturation, or plasma midazolam concentrations. It may represent a true regimen-related hemodynamic effect, but it may equally reflect residual confounding, heterogeneity in underlying cardiac diagnoses, baseline physiologic differences, measurement variability, or multiple testing across several physiologic endpoints. Larger studies reporting absolute MAP values and adjusting for cardiac diagnosis severity are needed to clarify this signal.

Previous studies have also reported blood pressure reductions after midazolam premedication. Masue et al. tested a high oral dose of midazolam (1.5 mg/kg) in infants and children presenting for cardiac surgery and found it to be a safe option without significant cardiovascular deterioration, although systolic arterial pressure reduction was among the more prominent hemodynamic findings [25]. Vasakova et al. [26] reported a similar reduction in systolic blood pressure 30 min after oral administration of midazolam for conscious sedation during dental procedures in children aged 1–12 years. These reports lend biological plausibility to a true, if modest, effect of midazolam on blood pressure; however, given the isolated nature of the finding and the absence of any clinical consequence in our cohort, we still argue for cautious interpretation.

Overall, the adverse effects of midazolam on oxygenation and hemodynamic stability appear more pronounced after intravenous administration for sedation than after administration via non-intravenous routes such as oral, intranasal, rectal, or sublingual [18,27].

This study has several strengths. It used a prospective randomized design in a clinically vulnerable pediatric cardiac population, accounted for administered doses in pharmacokinetic comparisons, and combined objective plasma concentrations with bedside behavioral and physiologic outcomes. The main limitations are its single-center design, modest sample size, incomplete pharmacokinetic data in three oral cases, heterogeneity in underlying cardiac diagnoses (cyanotic and acyanotic), and limited ability to adjust for disease severity or concomitant medications, along with the exploratory nature of multiple physiologic and behavioral comparisons. We acknowledge that with the distinctly different administration techniques used in the two groups, as described in the Methods section, it was not possible to ensure that the entire sublingual dose remained beneath the tongue throughout the absorption period, particularly in younger children. However, several measures were taken to maximize sublingual exposure. First, because the selected sublingual dose (0.3 mg/kg) was lower than the oral dose (0.5 mg/kg), the administered volume was correspondingly smaller, increasing the likelihood that at least a proportion of the drug remained in contact with the sublingual mucosa and was absorbed transmucosally. Second, in older and cooperative children, age-appropriate instructions were given to avoid immediate swallowing after administration, thereby prolonging the mucosal contact time. Nevertheless, partial swallowing in the sublingual group cannot be excluded, and four children who rejected the oral drug were excluded from the analysis. Both of these considerations reflect challenges inherent to drug administration in pediatric patients, rather than limitations specific to our study. Importantly, this reflects real-world clinical practice with a defensible and clinically useful finding: a lower nominal sublingual dose (0.3 mg/kg) achieved systemic exposure and sedation, parental separation, and mask acceptance outcomes comparable to those with the established oral dose (0.5 mg/kg), without worse heart rate or oxygen saturation responses. This study was also not powered to detect rare adverse effects or small-to-moderate differences in categorical behavioral outcomes. Finally, the study was registered retrospectively (April 2026), after recruitment had taken place. Although the clinical outcomes and statistical analyses were specified before the clinical data were collected and during initial project approval by the ethics committee, we acknowledge this genuine methodological limitation.

Determining optimal preanesthetic medication for pediatric patients remains an important yet unresolved challenge in perioperative care. Appropriate premedication can reduce anxiety, ease separation from parents, and enhance cooperation during anesthetic induction, thereby promoting a safer and more compassionate perioperative experience. In addition to its pharmacologic effectiveness, the premedication’s acceptability is also an important consideration [28]. An ideal agent should not only be pharmacologically effective but also be administered via a route readily accepted by children. Spitting, refusal, or incomplete intake of oral premedication is not uncommon in children. In an effort to predict uncooperative behavior in children, Lena et al. identified factors projecting anxiety and distress in children during anesthesia induction. Factors such as age between 1 and 3 years, parental anxiety, previous hospitalization, and previous operating theater experience—characteristics frequently encountered in pediatric cardiac patients—may increase the likelihood of resistant behavior [29]. The sublingual route may be a useful alternative and could support future child-friendly formulations. Kumar et al. evaluated sublingual midazolam delivered with an atomizer spray in children, while Hirokawa et al. reported successful sublingual midazolam premedication using a suction toothbrush in an adult with intellectual disabilities [30,31]. Compared with intranasal and rectal administration, sublingual administration may be preferable in some contexts because intranasal administration can cause nasal irritation and rectal administration may be uncomfortable or unacceptable [18].

Overall, our findings support oral and sublingual administration as clinically workable premedication strategies in this setting. In addition to comparing effectiveness, this study provides preliminary support for the further development of alternative sublingual formulations, including individualized 3D-printed medicinal films for pediatric use, with the goal of reducing procedural stress and improving patient acceptance [32].

Three-dimensional printing has been proven to be a feasible manufacturing prospect for drug-loaded films with promising dose, geometry, and physicochemical property control that may lead to potent, patient-tailored formulations. Because this method was not used in the present study, this possibility is noted only as a direction for future research, and no conclusions regarding 3D-printed formulations can be drawn from the current data [33,34,35].

## 5. Conclusions

In this single-center prospective randomized study of children with congenital heart disease, no statistically significant between-regimen differences in single-time-point plasma midazolam or 1-hydroxymidazolam concentrations, MAP, HR, SpO_2_, or behavioral outcomes remained after Holm correction. Sublingual administration was associated with a greater reduction in MAP at 30 min, a finding that should be interpreted cautiously and confirmed in larger studies. These results support the clinical feasibility of sublingual midazolam as a lower-dose premedication strategy and provide a rationale for further development of child-friendly sublingual formulations.

## Figures and Tables

**Table 1:** Baseline characteristics of the complete-case cohort.

Characteristic	Oral (O), n = 30	Sublingual (S), n = 35	*p*-Value
Age, years (mean ± SD)	3.13 ± 2.62	2.65 ± 2.24	0.532
Age, years [median (IQR)]	2.60 (0.85–4.69)	2.01 (0.69–4.80)	
Weight, kg (mean ± SD)	14.66 ± 6.38	15.51 ± 8.64	0.854
Weight, kg [median (IQR)]	12.00 (9.40–18.75)	13.90 (9.00–20.50)	
**Operation setting**			
Cardiac surgery	26 (86.7%)	32 (91.4%)	
Catheterization lab	4 (13.3%)	3 (8.6%)	

**Table 2:** Procedure categories in the complete-case cohort.

Procedure Type	Oral, n (%)	Sublingual, n (%)
ASD	7 (23.3%)	8 (22.9%)
ASD device closure	1 (3.3%)	0 (0.0%)
AV canal	2 (6.7%)	2 (5.7%)
Aortic arch/vascular ring	2 (6.7%)	3 (8.6%)
Complex cyanotic/staged repair	3 (10.0%)	2 (5.7%)
Electrophysiology/pacemaker	2 (6.7%)	4 (11.4%)
Other cardiac	4 (13.3%)	6 (17.1%)
PDA	1 (3.3%)	1 (2.9%)
VSD/conotruncal VSD	8 (26.7%)	9 (25.7%)

**Table 3:** Plasma concentrations.

Substance	Oral Mean ± SD	Sublingual Mean ± SD	Oral Median (IQR)	Sublingual Median (IQR)	Adjusted Ratio S/O (95% CI)	*p*	Holm-Adjusted *p*
Midazolam (ng/mL)	102.52 ± 61.98	75.04 ± 46.48	83.00 (54.15–141.50)	66.50 (36.95–100.60)	0.98 (0.53–1.79)	0.940	1.000
1-OH Midazolam (ng/mL)	40.04 ± 32.30	40.00 ± 43.89	31.55 (16.52–45.25)	24.10 (12.25–46.60)	1.37 (0.55–3.41)	0.494	1.000

**Table 4:** Hemodynamic and oxygenation changes from baseline.

Outcome	Oral Mean ± SD	Sublingual Mean ± SD	Adjusted Diff S−O (95% CI)	*p*-Value	Holm-Adjusted *p*
ΔMAP at 15 min, (mmHg)	1.27 ± 13.65	−3.80 ± 14.80	−6.08 (−13.57 to 1.40)	0.109	1.000
ΔMAP at 30 min, (mmHg)	5.34 ± 15.62	−5.42 ± 13.93	−12.08 (−19.74 to −4.42)	0.002	0.012
ΔHR at 15 min, (bpm)	−3.10 ± 17.70	−3.40 ± 10.95	−0.37 (−7.99 to 7.25)	0.923	1.000
ΔHR at 30 min, (bpm)	−2.77 ± 15.36	0.74 ± 13.27	3.34 (−4.09 to 10.76)	0.372	1.000
ΔSpO_2_ at 15 min, (%)	−0.77 ± 2.06	−0.63 ± 3.80	0.10 (−1.55 to 1.74)	0.907	1.000
ΔSpO_2_ at 30 min, (%)	−0.20 ± 6.07	0.88 ± 2.77	0.73 (−1.69 to 3.15)	0.549	1.000

**Table 5:** Behavioral outcomes.

Outcome	Oral Positive n/N (%)	Sublingual Positive n/N (%)	Odds Ratio S vs. O (95% CI)	*p* (Fisher)	Holm-Adjusted *p*
Positive 15 min score	24/30 (80.0%)	30/35 (85.7%)	0.67 (0.18–2.45)	0.742	1.000
Positive sedation rating	26/30 (86.7%)	31/35 (88.6%)	0.84 (0.19–3.69)	1.000	1.000
Easy separation from parents	25/30 (83.3%)	30/35 (85.7%)	0.83 (0.22–3.21)	1.000	1.000
Easy mask acceptance	13/30 (43.3%)	19/35 (54.3%)	0.64 (0.24–1.72)	0.459	1.000

## Data Availability

The data supporting this study are not publicly available due to national and institutional GDPR restrictions but are available from the corresponding author upon reasonable request for scientific purposes.

## Supplementary Materials

The following supporting information can be downloaded at: https://www.mdpi.com/article/10.3390/children13081067/s1, Table S1: Absolute hemodynamic and oxygenation values at baseline, 15, and 30 min.
