# Supplementary material for: Premedication of Pediatric Cardiac Population with Midazolam: Comparison of Oral and Sublingual Administration Regarding Plasma Midazolam Concentration, Clinical Effectiveness, Hemodynamic and Behavioral Outcomes
Source: Children (Basel). 2026 Aug 11;13(8):1067. doi: 10.3390/children13081067 (PMC13511163; doi:10.3390/children13081067)
Supplement: Supplementary file 1 [file children-13-01067-s001.zip › children-4424699-supplementary.pdf]

Table S1: Absolute hemodynamic and oxygenation values at baseline, 15, and 30 min.

**Outcome**	**Oral mean ± SD**	**Sublingual mean ± SD**
Absolute MAP at baseline, mmHg	68.59 ± 10.49	74.21 ± 11.97
Absolute MAP at 15 min, mmHg	68.39 ± 10.89	70.64 ± 11.34
Absolute MAP at 30 min, mmHg	74.17 ± 12.43	70.59 ± 12.05
Absolute HR at baseline, bpm	106.57 ± 21.26	106.54 ± 22.40
Absolute HR at 15 min, bpm	103.47 ± 18.44	103.14 ± 23.97
Absolute HR at 30 min, bpm	103.80 ± 15.58	107.29 ± 21.96
Absolute SpO_2 at baseline, %	95.03 ± 6.61	96.11 ± 4.96
Absolute SpO_2 at 15 min, %	94.27 ± 6.94	95.49 ± 7.86
Absolute SpO_2 at 30 min, %	94.83 ± 10.75	97.00 ± 6.48
